# Trans‐specific polymorphism and the convergent evolution of supertypes in major histocompatibility complex class II genes in darters (*Etheostoma*)

**DOI:** 10.1002/ece3.8485

**Published:** 2022-01-13

**Authors:** Kara M. Million, Curtis M. Lively

**Affiliations:** ^1^ Department of Biology Indiana University Bloomington Indiana USA

**Keywords:** convergent evolution, darters, MHC, supertypes, trans‐specific polymorphism

## Abstract

Major Histocompatibility Complex (MHC) genes are one of the most polymorphic gene groups known in vertebrates. MHC genes also exhibit allelic variants that are shared among taxa, referred to as trans‐specific polymorphism (TSP). The role that selection plays in maintaining such high diversity within species, as well as TSP, is an ongoing discussion in biology. In this study, we used deep‐sequencing techniques to characterize MHC class IIb gene diversity in three sympatric species of darters. We found at least 5 copies of the MHC gene in darters, with 126 genetic variants encoding 122 unique amino acid sequences. We identified four supertypes based on the binding properties of proteins encoded by the sequences. Although each species had a unique pool of variants, many variants were shared between species pairs and across all three species. Phylogenetic analysis showed that the variants did not group together monophyletically based on species identity or on supertype. An expanded phylogenetic analysis showed that some darter alleles grouped together with alleles from other percid fishes. Our findings show that TSP occurs in darters, which suggests that balancing selection is acting at the genotype level. Supertypes, however, are most likely evolving convergently, as evidenced by the fact that alleles do not form monophyletic groups based on supertype. Our research demonstrates that selection may be acting differently on MHC genes at the genotype and supertype levels, selecting for the maintenance of high genotypic diversity while driving the convergent evolution of similar MHC phenotypes across different species.

## Graphical abstract

In this study, we used deep‐sequencing techniques to characterize MHC class IIb gene diversity in three sympatric species of darters. We found at least 5 copies of the MHC gene in darters, with 126 genetic variants encoding 122 unique amino acid sequences, and we identified four supertypes based on the binding properties of proteins encoded by the sequences. Our findings show that trans‐specific polymorphism occurs in darters, which suggests that balancing selection is acting at the genotype level. Supertypes, however, are most likely evolving convergently, as evidenced by the fact that alleles do not form monophyletic groups based on supertype.

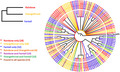

## 1 INTRODUCTION

Major histocompatibility complex (MHC) genes are an important component of the adaptive arm of the vertebrate immune system (Klein, 1986), and they are among the most diverse gene groups known in vertebrates. Thousands of MHC genetic variants are known to exist in humans alone (Beck & Trowsdale, 2000). Additionally, many taxa exhibit copy number variation (CNV) in MHC genes. Some species, such as passerine birds (Bollmer et al., 2010) and African cichlids (Hofmann et al., 2017), can have over a dozen copies of an MHC gene. The possession of multiple gene copies allows individuals to have different MHC genetic variants in their genotypes, allowing for high individual‐level diversity in this gene group.

Identical or highly similar MHC genetic variants also exist across taxa, a phenomenon known as trans‐specific polymorphism (TSP) (Arden & Klein, 1982; Klein, 1987). Not only has TSP been observed in MHC genes (Cutrera & Lacey, 2007; Dixon et al., 1996; Hoelzel et al., 1999; Moreno‐Santillán et al., 2016; Ono et al., 1992; Suárez et al., 2011), but it has also been found in other immune system‐related genes (Cagliani et al., 2010, 2012; Cortázar‐Chinarro et al., 2020; Su & Nei, 1999). TSP has also been observed in fungal genes (Devier et al., 2009; van Diepen et al., 2013) and in plant genes (Bouillé & Bousquet, 2005), including in plant self‐incompatibility genes (Nowak et al., 2011; Sutherland et al., 2008; Wang et al., 2020) and resistance genes (R‐genes) (Bechsgaard et al., 2017; Koenig et al., 2019). Host–pathogen interactions likely select for the maintenance of polymorphism within and across populations through overdominance and frequency‐dependent selection (Brockhurst et al., 2014; Hughes et al., 1994; Takahata & Nei, 1990). TSP can occur between closely related species (Meyer‐Lucht et al., 2008; Munguia‐Vega et al., 2007), but it can also occur across highly diverged vertebrate taxa (Mayer et al., 1992). Researchers have hypothesized that selection may be operating on MHC genes in multiple ways at multiple levels, which would explain why there is selection for high diversity even while there is selection for the maintenance of certain genetic variants or phenotypes. Lighten et al. (2017) argue that in MHCs, balancing selection may be operating at the phenotype level while arms‐race dynamics select for high allele turnover at the genotypic level (Lighten et al., 2017). Charlesworth, by contrast, claims that arms races are ruled out by high diversity and TSP in MHC genes (Charlesworth, 2006).

The mechanisms that maintain these high levels of diversity in MHC genes remain an open question in biology. Several hypotheses have been proposed concerning pathogen‐mediated selection on MHC genes and mate choice behaviors that select for higher diversity in MHCs. While some support has been found for these hypotheses across multiple systems, other studies have yielded mixed results (Kubinak et al., 2011). The differing results in these studies could be attributed to differences in the life histories and reproductive traits of the focal taxa, as well as differences in the selection pressures in the environments of these taxa, especially pathogen‐mediated selection pressures. A comparative approach to MHC studies across closely related taxa with different life histories and reproductive traits could yield valuable insight into the ways that these types of differences can shape the maintenance of MHC diversity across vertebrates.

In this study, we characterized immunogenetic diversity in three species of Darter (*Percidae*: Etheostomatini) that co‐occur in a single stream. Darters are a highly diverse group of freshwater fishes native to North America. The focal genus of our study, *Etheostoma*, is approximately 34 million years old, and it is estimated that the majority of extent darter species emerged within the past 15 million years (Near et al., 2011). Not only are darters highly speciose—more than 200 species have been described to date (Page & Burr, 2011)—but they also exhibit a diverse array of life histories and reproductive traits and behaviors (Kuehne & Barbour, 1983; Page, 1983). Darters are also host to multiple pathogens and parasites, some of which are generalists and some of which are highly specialized (Hoffman, 1999). One parasite group, monogeneans of the genus *Aethycteron*, prefers to infect darters in particular, and they demonstrate extremely high host specificity; each known parasite species in this genus is known to infect only one or (rarely) two host species (Beverley‐Burton & Klassen, 1990; Hoffman, 1999; Suriano & Beverley‐Burton, 1982). Darter species that occur in the same habitat can present with substantially different parasite burdens (Hanson & Stallsmith, 2013). This observation raises the question of whether different immunogenetic profiles within each species can explain the variation in susceptibility to local pathogens and parasites, especially to parasites that have specialized on, and are tightly co‐evolving with, their hosts. Darters are a promising system in which to study MHC gene diversity in vertebrates and to evaluate the mechanisms that maintain this diversity. Until this study, MHC diversity in darters had not yet been characterized or studied. Here, we use deep‐sequencing techniques to study the MHC class IIb gene in three species that occur in the same habitat while differing in their life histories and interactions with local parasites.

Our three focal species (*Etheostoma caeruleum*, *Etheostoma flabellare*, and *Etheostoma spectabile*) represent two subgenera (*E*. *flabellare* is in the subgenus *Catonotus* and *E*. *caeruleum* and *E*. *spectabile* are in the subgenus *Oligocephalus*). The two species in *Oligocephalus* are not sister species (Lang & Mayden, 2007; Near et al., 2011), although *E*. *caeruleum* and *E*. *spectabile* are known to hybridize when they co‐occur (Moran et al., 2017). *Etheostoma flabellare* is in a different subgenus, does not currently have genetic contact with the other two species, and has not done so for millions of years (Near et al., 2011). All three focal species co‐occur throughout much of their ranges (Page & Burr, 2011). While *E*. *caeruleum* and *E*. *spectabile* have highly similar microhabitat preferences, life history characteristics, and reproductive traits, *E*. *flabellare* substantially differs from the other two species across all these categories (Page, 1983; Page & Burr, 2011). This provides us the opportunity to study immunogenetic population characteristics in co‐occurring species that vary in how closely related they are and in the similarity of their life history characteristics. We expected that the species would share highly similar, if not identical, MHC genetic variants. We also predicted that *E*. *caeruleum* and *E*. *spectabile*, due to being more closely related, would share more MHC variants with each other than with *E*. *flabellare* and that their population immunogenetic structures would be more similar. Based on prior characterization of MHC class II genes in several other species in the same family as darters (Faulks & Östman, 2016), we predicted that darters would have between 4 and 6 copies of the MHC class IIb gene.

## 2 MATERIALS AND METHODS

### 2.1 Field site and specimen collection

A scientific purposes license for this research was issued to KMM by the Indiana Department of Natural Resources. This research was carried out with the approval of the IU Bloomington Institutional Animal Care and Use Committee (BIACUC protocol #19‐003).

Our field collections took place at a single site in Clear Creek, Bloomington, Indiana (39.12118432195659, 86.53886585996088), between 2018 and 2020. We collected three species: The Fantail Darter (*E*. *flabellare*), the Rainbow Darter (*E*. *caeruleum*), and the Orangethroat Darter (*E*. *spectabile*). One hundred and nine *E*. *flabellare*, 146 *E*. *caeruleum*, and 61 *E*. *spectabile* were used in this study. Specimens were collected using a seine net and euthanized on‐site in MS‐222 (500 mg/L). Specimens were placed on ice and transported to the Lively lab at Indiana University. Species identifications were confirmed using distinguishing morphological characteristics (Simon, 2011). Standard length, mass, and sex (when discernible) were recorded for each specimen. A 3–5‐mm^2^ sample of fin tissue was removed from the anal or pectoral fin of each sample and stored in 95% ethanol or stored in tissue lysis buffer and frozen at −20°C. Samples were each assigned a unique serial number and deposited in the Lively lab.

### 2.2 DNA extraction and PCR

DNA was extracted from fin clip tissue using a Qiagen DNEasy Blood and Tissue kit. The extractions were performed according to the manufacturer's instructions with the following modifications: tissue was incubated in the lysis buffer overnight rather than for 1 h, and following incubation 8 μl of RNAse A (100 mg/ml, Qiagen) was added to each sample before proceeding with the extraction. Concentration and 260/280 ratios of extracted DNA were measured on a Take3 spectrophotometer (Biotek), and concentration and integrity were measured using a TapeStation instrument (Agilent).

### 2.3 Primer design

At the time we undertook this study, no reference genome of any *Etheostoma* species was available and there were no published sequences of the gene of interest for our target taxa. We designed custom primers for this project using sequences from species in the same family as our target taxa (*Percidae*). We obtained sequences via GenBank from two species in the family *Percidae* (*Sander lucioperca* and *Perca fluviatilis*) of the gene of interest (MHC class IIB, exon 2, which encodes the peptide binding region [PBR]). We aligned these sequences using BioEdit (Hall, 1999) and identified well‐conserved regions within the exon at the 5′ and 3′ ends. From these regions we then developed and optimized a pair of primers: Eth2_MHC2B_F: 5′ AACTCSWCTGADCTKAAGGACAT 3′ and Eth_MHC2B_R: 5′ ACATCAATGTTGTGTTTGCAG 3′. The primers amplify a 164‐bp region of the approximately 200 bp MHC class IIb exon 2 gene in multiple species of *Etheostoma*. While the use of primers nested within the exon prevented us from obtaining a sequence in the entire exon, doing so allowed us to avoid amplification problems associated with intron length variability. The custom primers included overhang adaptors (5′ ACACTCTTTCCCTACACGACGCTCTTCCGATCT 3′ and 5′ GTGACTGGAGTTCAGACGTGTGCTCTTCCGATCT 3′).

### 2.4 Library prep and Illumina sequencing

We used a deep‐sequencing approach to genotype the individuals at the gene of interest. This approach has been shown to accurately genotype individuals in cases where there were multiple MHC loci (Lighten et al., 2014). Library prep and sequencing were completed at the Center for Genomics and Bioinformatics at Indiana University. Two rounds of PCR were performed: one round to amplify the gene of interest and one round to add a unique pair of indices (NEB i501‐508 and i701‐712) and sequencing adaptors to both ends of the product using a Nextera XT Index Kit.

Sequencing was performed on an Illumina MiSeq platform using a MiSeq Nano V2 kit (500 cycle). All of the sequencing runs were paired end. Five sequencing runs were performed for this study. To measure between‐run repeatability, 6 samples were selected from the second run and added to the samples for the third run. To measure within‐run repeatability, 5 DNA samples from the same individual were independently extracted, amplified, and loaded into the same run for 4 runs. Library prep was performed independently on all replicates. Repeatability was calculated by measuring the extent to which the genotypes of replicates matched and averaging the percent matches across the set of replicates.

### 2.5 Demultiplexing and quality control

Sequencing products were demultiplexed using the command DADA2 in the software QIIME 2. For the forward read, the first 22 bases were removed (library and primer sequence) and the remaining bases were truncated at 160 bp. Then for the remaining bases, the expected number of errors was calculated by summing the chance of error at each base (as indicated by the quality score). If this was greater than 2.0, the read pair was discarded. This process was then repeated for the reverse read except the first 20 bases were removed and the remaining bases were truncated at 80 bp. The forward and reverse reads were then overlapped. If the reads did not have an overlap of at least 12 bases, the read pair was discarded. Chimeric sequences were detected and filtered out using DADA2 and again using the software VSEARCH. VSEARCH was run in de novo mode with default parameters. The reads and counts were used to identify chimeras. The sequences were further filtered by removing any sequences that made up less than 1% of at least one sample.

### 2.6 Genotyping and copy number estimates

We used an Excel macro developed by Lighten et al. (2014) to determine the genotype of each individual and to estimate the number of MHC class II loci present in darters. We used degree of change (DOC) models and cumulative sequencing depths to distinguish true genetic variants from sequencing artifacts or errors. We used the CNV models in the macro and the range of the number of genetic variants found in our samples to estimate the number of MHC class II loci in darters. Although we were unable to assign genetic variants to individual loci, for the sake of simplicity, we hereafter refer to identified genetic variants as “alleles.” Genotyping was performed manually for each sample using the Excel macro and the methods referenced above.

### 2.7 Supertype assignment

We translated the allele DNA sequences into amino acid sequences using the online program ExPASy Translate (Gasteiger et al., 2003). We then examined the sequences for stop codons or nonsense codons in order to detect any nonfunctioning pseudogenes. We used the BLAST protein search function (NCBI) to identify the products of the sequences and to confirm that the sequences code for the MHC class IIb peptide‐binding region in fish.

To assign the MHCs to functional groups known as supertypes (Doytchinova & Flower, 2005; Guan et al., 2005), we first used the program HyPhy (default settings: ratio of synonymous to nonsynonymous mutations, neighbor‐joining tree, Tamura‐Nei model, maximum likelihood statistical method) in the software MEGA (Tamura et al., 2013) to detect which of the 54 codons in the sequence were under positive selection (positively selected sites, or PSS). We then used five published physicochemical characteristics (Z1, Z2, Z3, Z4, and Z5) (Sandberg et al., 1998) for the positively selected amino acids in the sequences and performed a Discriminant Analysis of Principal Components (DAPC) using the program adegenet in R (Jombart, 2008; Jombart & Ahmed, 2011). The program uses the binding properties as morphological characteristics and assigns the variants into clusters (in our case, these clusters are the supertypes). We retained the number of principal components that accounted for the most cumulative variance based on the eigenvalues of the PCA. We then selected the cluster assignment with the lowest Bayesian Information Criterion.

### 2.8 Neighbor‐Joining trees

We used the software MEGA (Tamura et al., 2013) to construct and test neighbor‐joining trees describing the relationships between the alleles based on Jukes‐Cantor distances and with 1,000 bootstrap replicates. We condensed the trees down to the branches with bootstrap support of 50% or higher. We constructed an additional neighbor‐joining tree that included MHC IIb sequences from three additional non‐darter species in the family *Percidae* (*Sander vitreus*, *P*. *fluviatilis*, and *S*. *lucioperca*) (Abram et al., 2018; Faulks & Östman, 2016, Krause, Sale, and Dixon, 2016, direct submission) and from species in the families *Centrarchidae* (*Lepomis macrochirus*) (Silveira, 2019, Genbank direct submission), Poeciliidae (*Poecilia reticulata* (van Oosterhout et al., 2006), and *Gasterosteidae* (*Gasterosteus aculeatus*) (Lenz et al., 2009). For the latter tree, we used a sequence from the non‐teleost fish *Lepisosteus osseus* (Venkatesh et al., 1999) as an outgroup to root the tree. Accession numbers for sequences obtained from GenBank are available in Table S1.

## 3 RESULTS

Within‐run repeatability was 100%, and between‐run repeatability was 87%, indicating that the sequencing method is reliable for genotyping our species of interest (Lighten et al., 2014). The average number of reads obtained per sample was 4,205. We discarded any samples from further analysis that contained fewer than 100 reads.

One hundred and twenty‐seven putative alleles (PAs) were identified among the three species. One allele contained a stop codon at the end of the sequence, indicating that it may be a pseudogene. Another allele was short by one codon at the end of the sequence. We set these alleles aside from further analysis and focused on the remaining 125 PAs that contained no stop codons or nonsense codons, that matched fish MHC class IIb exon 2 sequences in NCBI BLAST nucleotide searches, and whose products matched fish MHC class II peptides in NCBI BLAST protein searches.

The CNV models and the range of PAs observed per individual in each species (1–10) indicate the presence of at least 4 copies of the MHC class II gene in *E*. *spectabile* and at least 5 copies in *E*. *flabellare* and *E*. *caeruleum* (Table 1). We could not assign individual alleles to loci due to the lack of a reference genome at the time we were executing this project. However, an annotated genome for *E*. *spectabile* has recently been published (Moran et al., 2020). A BLAST search revealed that our sequenced alleles matched 4 regions in the published genome that are predicted to code for the MHC class Iib peptide binding region. These results suggest that our estimated number of loci (4) for *E*. *spectabile*, at least, is accurate.

**TABLE 1:** Number of alleles per species and unique to each species, as well as the maximum number of alleles per individual and estimated number of loci in each species

Species	Total alleles in species	Total alleles unique to species	Maximum number of alleles per individual	Estimated number of loci
*Etheostoma caeruleum*	55	28	10	5
*Etheostoma flabellare*	65	32	9	5
*Etheostoma spectabile*	55	28	8	4

**FIGURE 1:**
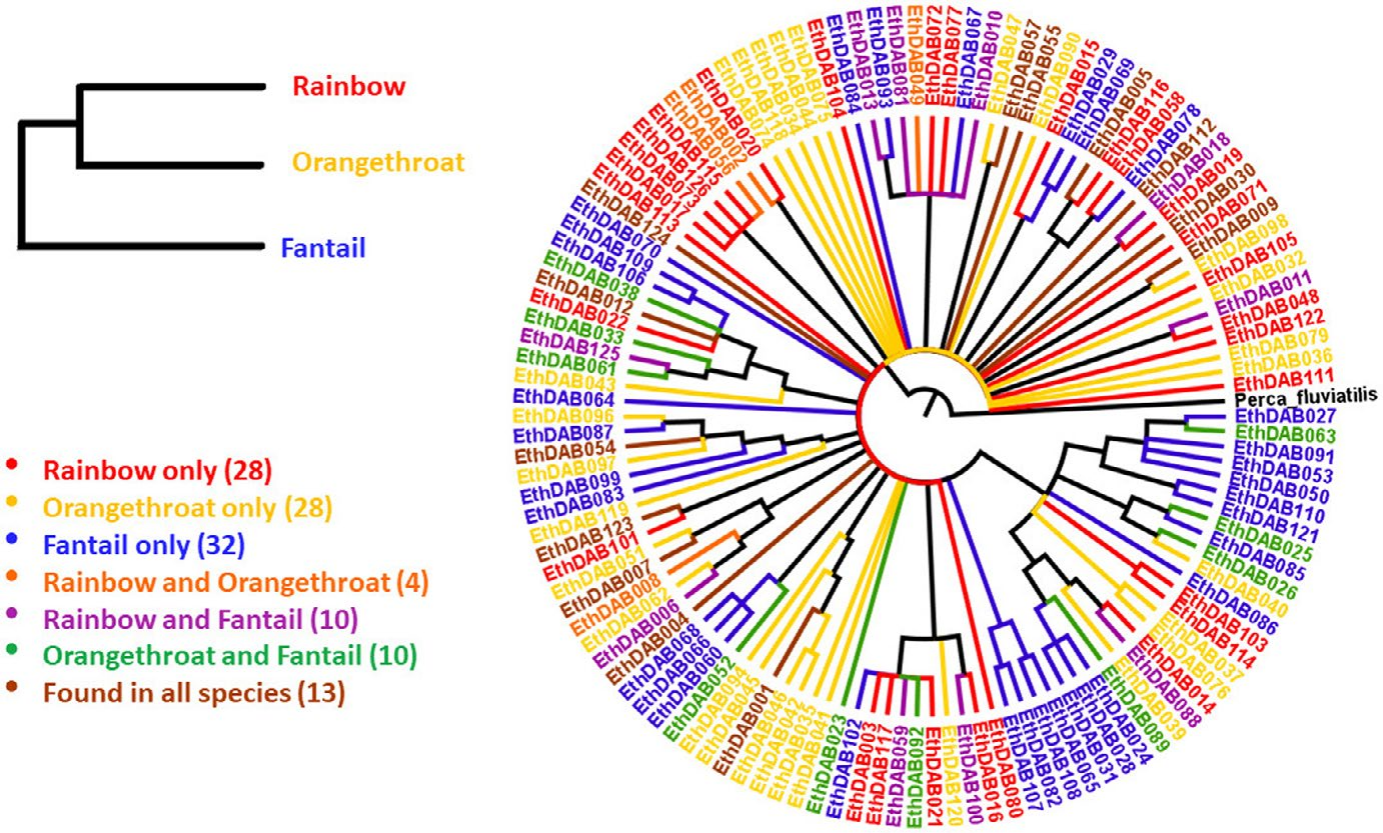
Neighbor joining phylogenetic analysis of MHC IIb genetic variants obtained from three darter species. Colors indicate the species in which each allele was found. Cladogram on the left represents the phylogenetic relationships between the three species. Outgroup *Perca fluviatilis* was used to root the tree

We observed that many alleles were shared between species. Thirteen alleles occurred across all three species, and each species pair had alleles shared exclusively between them and not with the third species (Figure 1). Additionally, a unique group of alleles within each species was observed. Therefore, although each species had a unique pool of alleles in its population, there were identical alleles that occurred in more than one species and across all three. The number of alleles unique to each species was comparable across *E*. *caeruleum*, *E*. *spectabile*, *a*nd *E*. *flabellare* (28, 28, and 32, respectively), as was the total number of alleles observed in each species (55, 55, and 65, respectively) (Table 1).

Although *E*. *caeruleum* and *E*. *spectabile* are more closely related to each other than either species is to *E*. *flabellare*, the two species shared fewer alleles between them (4) than either species shared with *E*. *flabellare* (10 and 10, respectively).

One hundred and twenty‐six alleles were coded for 122 unique amino acid sequences. Only two pairs of alleles coded for identical amino acid sequences (Figure 1), indicating that genetic diversity in these genes is a reasonable representation of phenotypic diversity in MHC class II peptides.

**FIGURE 2:**
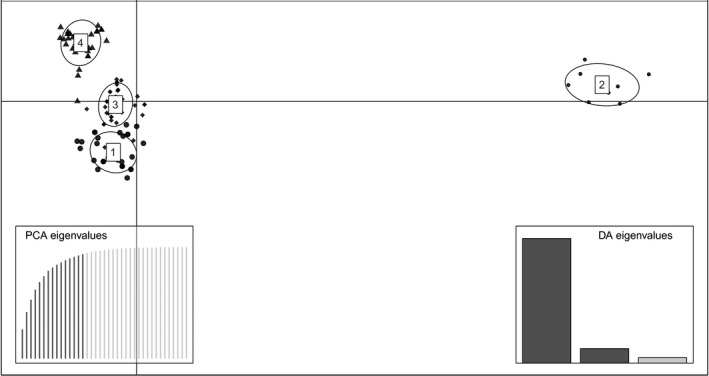
Discriminant analysis of principal components result in which all alleles are clustered into functional supertypes based on the binding properties of the encoded amino acids. Boxes on the left and right display the eigenvalues from the principal component analysis and the discriminant analysis, respectively

The observed alleles grouped into 4 distinct functional supertypes (Figure 2). All 4 supertypes occurred in all three species included in this study (Table 2). While three of the supertypes contained many alleles, one supertype (ST2) was only represented by 8 alleles.

**TABLE 2:** Frequencies at which each supertype (ST) occurs in each species and among all species in our study

	ST1	ST2	ST3	ST4
*Etheostoma caeruleum*	0.21	0.16	0.82	0.34
*Etheostoma flabellare*	0.76	0.16	0.27	0.47
*Etheostoma spectabile*	0.26	0.21	0.67	0.59
All	0.41	0.17	0.59	0.44

**FIGURE 3:**
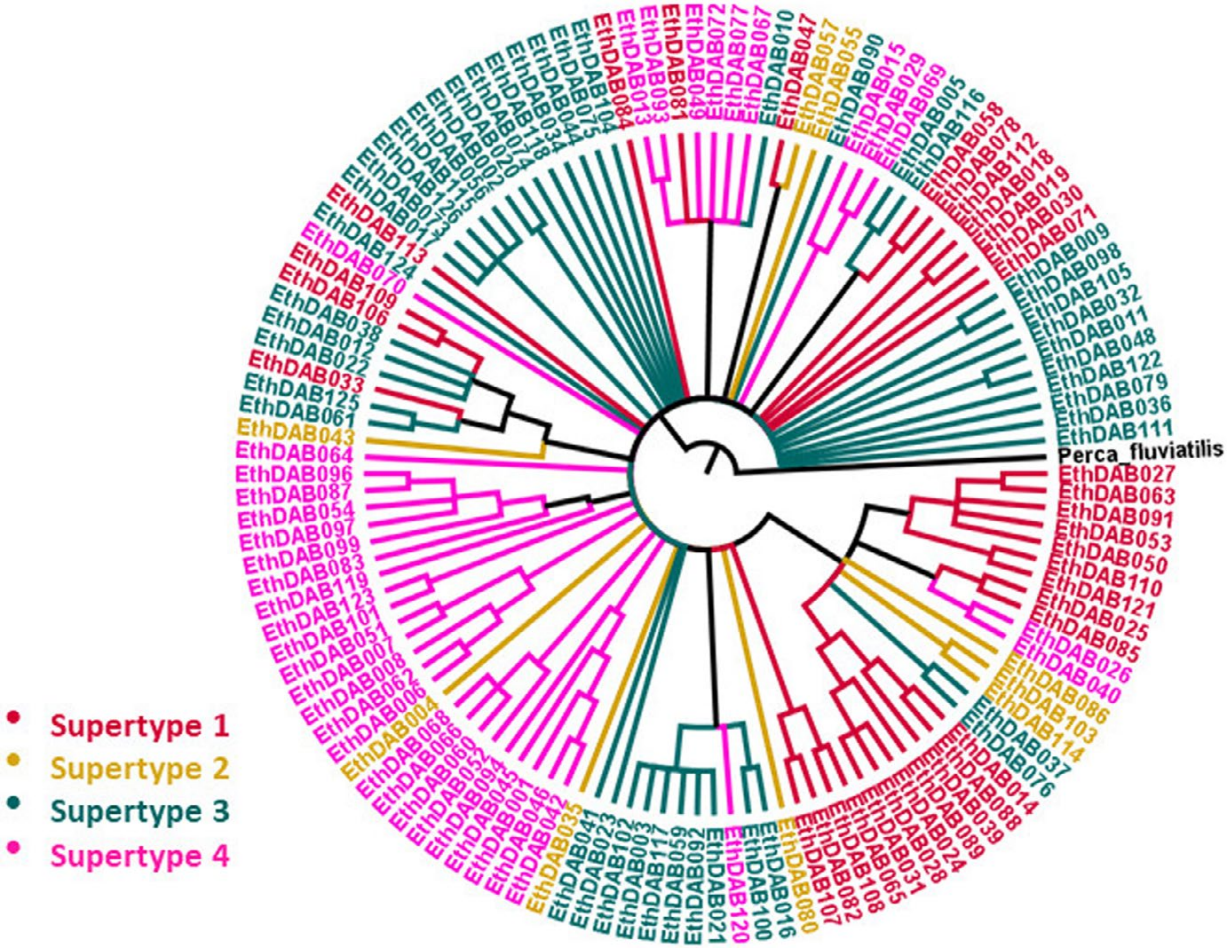
Neighbor joining phylogeny of darter MHC IIb genetic variants. Colors indicate the supertype into which each genetic variant was placed in the DAPC. Outgroup *Perca fluviatilis* was used to root the tree

There was no tendency for the alleles to group together phylogenetically based on the species in which they occurred (Figure 1). There was more of a tendency for the alleles to cluster together on the phylogeny based on supertype (Figure 3); however, none of the supertypes formed a monophyletic group.

**FIGURE 4:**
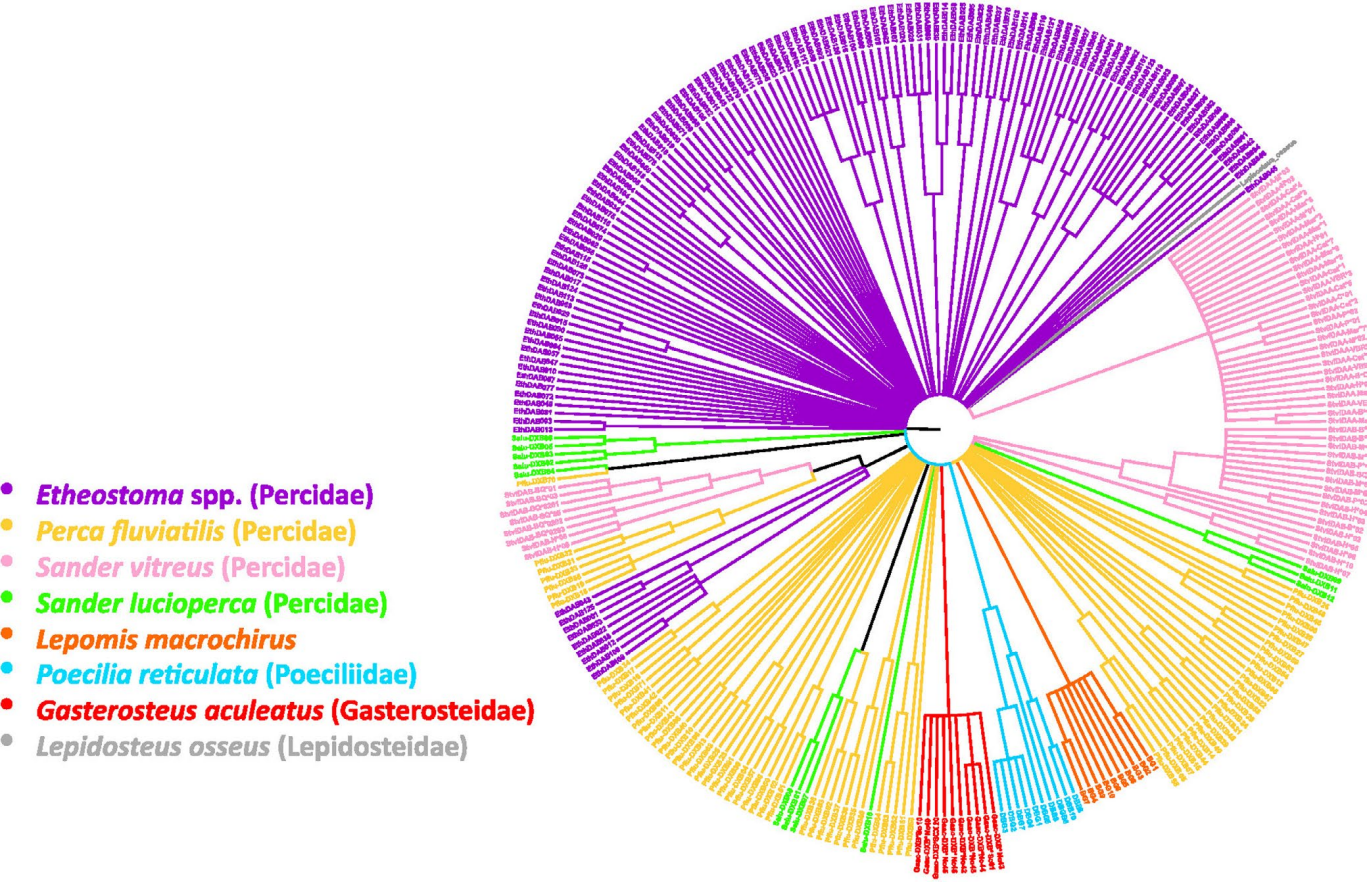
Neighbor joining phylogenetic analyses of MHC IIb alleles obtained from darters, three other species within Percidae, and three species in other families, with Longnose Gar (a non‐teleost fish) as an outgroup. Families are listed after each taxon in parentheses in the legend

On the multispecies neighbor‐joining tree (Figure 4), alleles from *L. macrochirus* formed a single species‐specific monophyletic clade with 99% bootstrap support, as did alleles from *P*. *reticulata* (87%) and *G*. *aculeatus* (88%). By contrast, none of the alleles from the species within *Percidae* formed a monophyletic clade containing all the alleles from a single species. All three of the non‐darter species in *Percidae* formed clades with each other several times. Some darter alleles occurred in a group that included alleles from *S*. *vitreus* and *P*. *fluviatilis*. In contrast to the alleles from all other taxa represented, many of the alleles from the darters were highly divergent and did not group together with any other alleles.

## 4 DISCUSSION

The estimated number of copies of the MHC class II gene was consistent with our expectations. Based on the number of copies in other species in *Percidae* (Faulks & Östman, 2016), we estimated that darters would have between 4 and 6 copies of this gene, and our sequencing results suggest they have 4–5 copies. The recent publication of an annotated genome for *E*. *spectabile* (Moran et al., 2020) allowed us to compare our results from *E*. *spectabile* to the published genome. Our findings are consistent with the number of loci predicted by the annotated genome. Our detection of the presence of 5 loci in two of our species of interest and 4 in *E*. *spectabile* supports the existence different numbers of gene copies between species. As more genomic resources become available for darters, future research can explore whether CNV occurs within darter species and the allelic variants that occur at specific loci.

Despite the possibility of having up to 10 distinct alleles per individual in two of our species and up to 8 in the third, most of the individuals we sequenced only had 2 or 3 alleles in their genotype. This observation could be explained by within‐species CNV in darters, but further research is needed to confirm such a hypothesis. Some studies have shown that there is selection pressure on MHC loci to have an optimal amount of variation, but not an excessively high amount (Kalbe et al., 2012). It is hypothesized that the accumulation and maintenance of too many alleles at these loci could incur a fitness cost, such as an overly active immune system that negatively impacts the individual's fitness. An optimal number of alleles would allow an individual to reap the advantages of a diverse immune system repertoire without incurring the potential costs (Wegner et al., 2003). Follow‐up research in darters could test whether individuals with extremely high numbers of alleles have overactive immune systems or incur other costs that affect their fitness compared to individuals with a moderate number of alleles. Until such research is conducted, the reason that most darters in these populations exhibit fewer alleles than the possible maximum of 10 remains an interesting open question.

Our findings indicate that genetic diversity is a fairly accurate representation of phenotypic diversity in darters for MHC class IIb genes. This observation has important implications for future research in which we seek to test competing hypotheses for the maintenance of MHC diversity in darters, particularly hypotheses that hinge on parasites interacting with the host immune system. We have confirmed that we can use genotype as a reasonable proxy for phenotype when testing hypotheses concerning these host–parasite interactions.

The fact that the three species share alleles between them and that the alleles do not group together phylogenetically based on the hosts in which they occur lead us to conclude that TSP is occurring in darters at the MHC class II gene. We considered the possibility that species could be sharing alleles due to introgression, a common occurrence in darters (Bossu & Near, 2009; MacGuigan & Near, 2019). However, the two more closely related species (*E*. *spectabile* and *E*. *caeruleum*) actually shared fewer alleles between them than either species shared with *E*. *flabellare*. Although the two more closely related species do occasionally hybridize (Moran et al., 2017), there has been no genetic contact between either species and *E*. *flabellare* for millions of years (Near et al., 2011). In fact, *E*. *flabellare* is in a different subgenus (*Catonotus*) than the other two species (*Oligocephalus*) (Page, 1983). Taken together, these observations lead us to conclude that trans‐species polymorphism is occurring across darter species in the MHC class Iib gene.

TSP is common in MHC genes. It is thought that it occurs because selection is maintaining alleles across species and preventing complete turnover (Hughes et al., 1994; Takahata & Nei, 1990). TSP could have implications for the way parasites interact with their hosts. For example, a generalist parasite that can infect all species as hosts will see a different immunogenetic landscape from a specialist parasite that only infects one host species. This might also explain why, when researchers have attempted to test parasite‐related hypotheses concerning the maintenance of MHC diversity, they have obtained conflicting results (Kubinak et al., 2011). The results could depend on the parasites/pathogens chosen to test the hypotheses, and on the population immunogenetic structure of the focal species as well as that of co‐occurring species that can share alleles and pathogens with the focal species.

Of the four supertypes we identified in darters, one supertype was represented by fewer alleles than the other three, and it was much more divergent from the others in the phenotype space generated by the DAPC. Supertypes are classified based on the function of the protein products (the binding properties). The fact that this supertype contains fewer genetic variants and is much more diverged phenotypically from the other three supertypes suggests that selection may be acting differently on this supertype than on the others. We aim to evaluate this topic in more depth as we explore immunogenetic diversity in this system in future research.

It has been hypothesized that selection could act differently on supertypes than on genotypes (Lighten et al., 2017). If this were true, it could affect the way parasites interact with the host immune system. All four supertypes were represented in all three darter species, suggesting that polymorphism is being maintained at the supertype level as well as the genotype level. Lighten et al. (2017) argue that selection can act differently at the genotype level and the supertype level: balancing selection maintains supertype polymorphism while within supertypes positive selection drives down allelic diversity and causes high allelic turnover. They refer to their analysis of guppy MHC class II genotype and supertype diversity and simulations of supertypes evolving in paratope space as support for their argument. Ejsmond et al. (2018), however, state that under the scenario Lighten et al. assert (balancing selection maintains supertypes while alleles experience rapid turnover), several other predictions should be met: supertype lineages should be monophyletic and sharing of identical alleles between species should be rare. In our case none of the identified supertypes form monophyletic groups, and many alleles (30%) are shared between species. Our findings are similar to those of Lighten et al. for guppies, but we are inclined to agree with the interpretation of Ejsmond et al.: that supertypes are evolving across species through convergence and that their role in maintaining TSP, if any, remains an open question. Our results indicate that TSP is occurring in darters, but it is not primarily being driven by the maintenance of supertype lineages.

Our conclusions are limited by the fact that our genotyping methods captured most, but not all, of the gene of interest. Future research will use newly available genomic resources for darters to gain a more complete view of the variability present in this gene.

Not only does our work set us up to test hypotheses concerning the maintenance of MHC diversity in darters, but our development of these methods in darters could have conservation applications as well. Research shows that many attempts to propagate and re‐introduce endangered species into their native habitats fail (Frankham et al., 2002). One possible reason for this failure is that captive‐bred populations are not immunogenetically adapted to local parasites and pathogens in the wild (Kubinak et al., 2011). Many species of darter are threatened or endangered in the wild. Several institutions focus on maintaining ark populations of these species and propagating darters for release into their native ranges. Although markers have been developed to monitor genetic diversity in captive darter populations (Tonnis, 2006), the addition of immunogenetic markers can enable conservation scientists to better adapt captive‐bred populations to disease pressures in the wild.

The maintenance of diversity in MHC genes is an interesting question in vertebrate evolution. The key to answering this question may lie in the study of immunogenetics across a wide range of vertebrate taxa, especially in groups well‐suited for comparative analyses (Bernatchez & Landry, 2003). Our work has shown that selection may be maintaining high genotypic diversity in MHC genes, while selecting for the convergent evolution of similar phenotypes (MHC functional supertypes) across species.

## CONFLICT OF INTEREST

The authors declare no conflict of interest.

## AUTHOR CONTRIBUTIONS

**Kara M. Million:** Conceptualization (lead); data curation (lead); formal analysis (lead); funding acquisition (lead); investigation (lead); methodology (lead); project administration (equal); resources (equal); software (lead); supervision (equal); validation (equal); visualization (lead); writing – original draft (lead); writing – review and editing (equal). **Curtis M. Lively:** Conceptualization (supporting); data curation (supporting); formal analysis (supporting); funding acquisition (supporting); investigation (supporting); methodology (supporting); project administration (supporting); resources (equal); supervision (equal); writing – original draft (supporting); writing – review and editing (equal).

## Supporting information

Table S1

## Data Availability

Data associated with this manuscript have been deposited in Dryad at https://doi.org/10.5061/dryad.t76hdr82h.
