# Supplementary material for: Trans‐specific polymorphism and the convergent evolution of supertypes in major histocompatibility complex class II genes in darters (Etheostoma)
Source: Ecol Evol. 2022 Jan 13;12(1):e8485. doi: 10.1002/ece3.8485 (PMC9601779; doi:10.1002/ece3.8485)
Supplement: Supplementary file 1 — Table S1 [file ECE3-12-e8485-s001.docx]

| Species | Genbank accession number(s) |
| --- | --- |
| *Gasterosteus aculeatus* | FJ360532.1-FJ360541.1 |
| *Lepisosteus osseus* | AF134922.1 |
| *Lepomis machrochirus* | MK620857.1-MK620866.1 |
| *Perca fluviatilis* | KT865110.1- KT865180.1 |
| *Poecilia reticulata* | AY747137.1-AY747146.1 |
| *Sander lucioperca* | KT865181.1-KT865192.1 |
| *Stizostedion vitreum* | AY158839.1-AY158863.1, AY158874.1-AY158884.1, AY364411.1-AY364429.1 |

Supplemental Table S1. Genbank accession numbers of sequences obtained for phylogenetic analysis.
